# Supplementary material for: Thioredoxin interacting protein (TXNIP), a redox regulator, mediates the EPAC-RAP1 signaling dependency of primary melanoma
Source: Oncogene. 2026 Jul 25;45(35):3702–18. doi: 10.1038/s41388-026-03895-6 (PMC13503283; doi:10.1038/s41388-026-03895-6)
Supplement: Supplementary file 2 [file 41388_2026_3895_MOESM2_ESM.pdf]

Antibodies	Catalog#	Company
Cyclin E1 (HE12) Mouse mAb	4129	Cell signaling
Ki67 Rabbit mAb	ab16667	Abcam
S100A4 (D9F9D) Rabbit mAb	13018	Cell signaling
Rat IgG	A21209	Thermo Fisher
TXNIP (D5F3E) Rabbit mAb	14715	Cell Signaling
Thioredoxin 1 (C63C6) Rabbit mAb	2429	Cell Signaling
CD8 Rat mAb	14-0808-82	Invitrogen
CD4 Rat mAb	14-9766-82	Invitrogen
HIF-1α (D1S7W) XP® Rabbit mAb	36169	Cell Signaling
ARRDC4 Polyclonal Antibody	PA5-99591	Thermo Fisher
Rap1-GTP Mouse mAb	26912	new east
p21 (12D1) Rabbit mAb	2947	Cell Signaling
PTEN (138G6) Rabbit mAb	9559	Cell Signaling
Rap1A/Rap1B Rabbit pAb	4938	Cell Signaling
Phospho-Akt -ser473 Rabbit pAb	9271	Cell Signaling
Akt Rabbit pAb	9272	Cell Signaling
GAPDH Mouse mAb	60004-1-Ig	Proteintech
Axl (C89E7) Rabbit mAb	8661	Cell Signaling
E-Cadherin (24E10) Rabbit mAb	3195	Cell Signaling
N-Cadherin (D4R1H) XP® Rabbit mAb	13116	Cell Signaling
MITF (D5G7V) Rabbit mAb	12590	Cell Signaling
mTOR (7C10) Rabbit mAb	2983	Cell Signaling
Phospho-mTOR (Ser2448) Antibody	2971	Cell Signaling
BRAF (V600E) Rabbit mAb	SAB5600047	Millipore Sigma
EPAC1 Rabbit pAb	12572-1-AP	Proteintech
EPAC2 Rabbit pAb	19103-1-AP	Proteintech
Anti-rabbit IgG, HRP-linked Antibody	7074	Cell Signaling
Anti-mouse IgG, HRP-linked Antibody	7076	Cell Signaling
β-tubulin (9F3) Rabbit mAb	2128	Cell Signaling
p16 INK4A (D7C1M) Rabbit mAb	80772	Cell Signaling
CDK2 (78B2) Rabbit mAb	2546	Cell Signaling
Cyclin B1 (D5C10) XP® Rabbit mAb	12231	Cell Signaling
p27 Kip1 (D69C12) Rabbit mAb	3686	Cell Signaling
Cyclin D1 Rabbit pAb	2922	Cell Signaling
Cyclin A2 (BF683) Mouse mAb	4656	Cell Signaling
CDK4 (D9G3E) Rabbit mAb	12790	Cell Signaling
DDK (D6W5B) Rabbit mAb	14793	Cell Signaling
p44/42 MAPK (Erk1/2) (L34F12) Mouse mAb	4696	Cell Signaling
Phospho-p44/42 MAPK (Erk1/2) (Thr202/Tyr204) (D13.14.4E) Rabbit mAb	4370	Cell Signaling
Thioredoxin-2 (D1C9L) Rabbit mAb	14907	Cell Signaling

FoxO1 (C29H4) Rabbit mAb	2880	Cell Signaling
FoxO3 (D19A7) Rabbit mAb	12829	Cell Signaling
NRF2 (D1Z9C) Rabbit mAb	12721	Cell Signaling
MondoA (F8A8L) Rabbit mAb	72133	Cell Signaling
Chemicals	Catalog#	Company
Bovine Serum Albumin	A2153	Sigma
GoTaq® Master Mixes	M7122	Promega
Thermo Scientific™ Halt™ Protease Inhibitor Cocktail (100X)	PI78430	Fisher Scientific
4-Hydroxytamoxifen	SML1666-1ML	Sigma
Invitrogen™ 1 Kb Plus DNA Ladder	10-787-018	Fisher Scientific
Invitrogen™ Novex™ Sharp Pre-Stained Protein Standard	LC5800	Fisher Scientific
Invitrogen™ MagicMark™ XP Western Protein Standard	LC5602	Fisher Scientific
Lipofectamine 3000	L3000015	Invitrogen
Xylenes, Reagent ACS, 99%	AC422685000	Fisher Scientific
RIPA Lysis and Extraction Buffer	PI89901	Fisher Scientific
Restore™ PLUS Western Blot Stripping Buffer	PI46430	Fisher Scientific
DAPI	H-1800	Vector Labs
Gibco™ DMEM, high glucose, pyruvate	11-995-073	Fisher Scientific
Gibco™ Antibiotic-Antimycotic (100X)	15-240-096	Fisher Scientific
MCDB 153 Medium	M7403	Sigma
Gibco™ Fetal Bovine Serum, heat inactivated	16-140-071	Fisher Scientific
Gibco™ Fetal Bovine Serum	10-437-028	Fisher Scientific
254 medium	M154500	Fisher Scientific
HMGS	S0025	Fisher Scientific
Universal Mycoplasma PCR Detection Kit (ATCC)	50-238-3275	Fisher Scientific
Cell Proliferation Kit I (MTT)	11465007001	Sigma
Gibco™ Opti-MEM™ I Reduced Serum Medium	31-985-062	Fisher Scientific
Mouse and Rabbit specific HRP/DAB IHC Detection kit	ab236466	Abcam
Opal 7 color IHC Kit	NEL797B001KT	PerkinElmer
RNA extraction kit	74104	Qiagen
Donkey serum	AB7475	Abcam
High-Capacity cDNA Reverse Transcription Kit	4368814	Thermo Fisher
β-Galactosidase Staining Kit	9860	Cell Signaling
Lipofectamine RNAiMax transfection reagent	13778150	Thermo Fisher
Lipofectamine 3000	L3000015	Invitrogen
Polybrene Infection / Transfection Reagent	TR-1003	Sigma-Aldrich
Pierce™ ECL Western Blotting Substrate	32209	Thermo Fisher
Cell Biolabs Inc QuickTiter™ Lentivirus Titer Kit (Lentivirus-Associated p24 ELISA).	NC9651553	Fisher Scientific
Zymo Pure Plasmod Maxiprep Kit	NC0835051	Fisher Scientific
Dneasy Blood and Tissue kit	69504	Qiagen
TaqMan™ Fast Advanced Master Mix for qPCR	4444556	Thermo Fisher

CellROX™ Green Reagent	C10444	Thermo Fisher
**Chemicals**	**Catalog#**	**Company**
Rapamycin	S1039	Selleck Chemicals
Cobalt(II) chloride	232696-5G	Sigma
ESI-09	S7499	Selleck Chemicals
ESI-05	6320	Tocris
MG132	S2619	Selleck Chemicals
N-acetyl cysteine	S1623	Selleck Chemicals
Glutathione	S4606	Selleck Chemicals
MitoQ	S8978	Selleck Chemicals
Selumetinib (AZD6244)	S1008	Selleck Chemicals
Vemurafenib (PLX4032)	S1267	Selleck Chemicals
Rotenone	R8875	Sigma
KY12420(Wortmannin)	S2758	Selleck Chemicals
KRX0401(Perifosine)	S1037	Selleck Chemicals
**Plasmids**	**Catalog#**	**Company**
PTEN shRNA Plasmid (h)	sc-29459-SH	Santa Cruz
Rap1GAP shRNA Plasmid (h)	sc-36388-SH	Santa Cruz
Epac2 shRNA Plasmid (h)	sc-41702-SH	Santa Cruz
TXNIP (NM_006472)	RC210804	OriGene
p(HA)HIF1alpha (P402A,P564A)	52636	Addgene
**siRNA**		
siEPAC1	s20360, s20361	Thermo Fisher
siEPAC2	s21814, s21815	Thermo Fisher
Control siRNA (Fluorescein Conjugate)-A	sc-36869	Santacruz
**Taqman primers**	**Catalog#**	**Company**
RAPGEF3-FAM	Assay ID:Hs01030417_m1	Thermo Fisher
RAPGEF4-FAM	Assay ID:Hs00199754_m1	Thermo Fisher
TXNIP-FAM	Assay ID:Hs01006899_g1	Thermo Fisher
ARRDC4-FAM	Assay ID:Hs00411771_m1	Thermo Fisher
HIF-1a-FAM	Assay ID:Hs00153153_m1	Thermo Fisher
GAPDH-FAM	Assay ID:Hs02786624_g1	Thermo Fisher
**Genotyping primers**		
Epac1 WT	5'-CTGGCCTCTCCTGAATCTTG-3'	
	5'-CCTCGCTGTTGGTAAGTGGT-3'	
Epac1 KO	5'-AATGGGCTGACCGCTTCCTCGT-3'	
	5'-GCCATAGCCTCAACAAGCTC-3'	
Epac2 WT	5'-CCTCCCTTTTGCTCTCTCCT-3'	
	5'-CGCTCGCTGCATTTGTATTA-3'	
Epac2 KO	5'-AATGGGCTGACCGCTTCCTCGT-3'	
	5'-CGCTCGCTGCATTTGTATTA-3'	

Cell Lines	Source
WM115	Rockland Immunochemicals
WM165-1	Rockland Immunochemicals
WM266-4	Rockland Immunochemicals
WM239-A	Rockland Immunochemicals
WM1552C	Rockland Immunochemicals
WM1862	Rockland Immunochemicals
WM35	Rockland Immunochemicals
WM75	Rockland Immunochemicals
MRA2	Gifted by Dr. Mark Albertini (UW-Madison)
MRA5	Gifted by Dr. Mark Albertini (UW-Madison)
MRA6	Gifted by Dr. Mark Albertini (UW-Madison)
MRA9	Gifted by Dr. Mark Albertini (UW-Madison)
MRA12	Gifted by Dr. Mark Albertini (UW-Madison)
MRA13	Gifted by Dr. Mark Albertini (UW-Madison)
